# Coronary Microvascular Dysfunction in Stress Cardiomyopathy: At the Heart of the Problem

**DOI:** 10.3390/life16071091

**Published:** 2026-06-29

**Authors:** Giorgio Piccolboni, Giovanni Civieri, Francesco Tona

**Affiliations:** 1 Cardiology Division, Department of Cardiac, Thoracic, Vascular Sciences and Public Health, University of Padua, 35128 Padua, Italy; giorgio.piccolboni@studenti.unipd.it (G.P.); giovanni.civieri@gmail.com (G.C.); 2 PhD Program in Translation Specialistic Medicine “G.B. Morgagni”, Curriculum “Cardiovascular Sciences”, University of Padua, 35128 Padua, Italy

**Keywords:** Takotsubo syndrome, sympathetic hyperactivation, inflammation, coronary microvascular disfunction

## Abstract

Takotsubo syndrome (TTS) is an acute disorder characterized by transient left ventricular dysfunction with typical regional wall motion abnormalities, most commonly apical ballooning. It accounts for 1–3% of all suspected acute coronary syndromes and up to 5–6% in women presenting with ST-segment elevation myocardial infarction requiring coronary angiography to exclude obstructive coronary artery disease. The pathophysiology of TTS is complex and not fully elucidated, with sympathetic hyperactivation playing a central role through calcium dysregulation, oxidative stress, and metabolic alterations. Both clinical and experimental data demonstrate the importance of inflammation, with cell infiltration and persistent immune activation exceeding the acute phase. Increasing evidence highlights the impact of coronary microvascular disfunction (CMVD) as a secondary phenomenon, with some findings that support its role as a causative substrate. Beyond well-known predisposing conditions such as female sex, postmenopausal age, and neurological and psychiatric disorders with the trigger of a physical or psychological event, numerous case reports associate the syndrome with chronic autoimmune diseases, even if clear experimental evidence remains poor and worthy of further study. Echocardiography and advanced imaging techniques, including cardiac magnetic resonance and positron emission tomography, have provided insights into transient CMVD, reversible myocardial edema, and metabolic impairment, strengthening our knowledge of the syndrome as a dynamic process. It is also of growing interest to perform invasive hemodynamic assessment to explain the increase in microvascular resistance. This review offers a comprehensive and up-to-date overview of these techniques in the context of TTS. Since clinically, TTS may be associated with significant morbidity and mortality, with some unexplained cases of long-term myocardial disfunction or even recurrence, a deeper understanding of the interplay between catecholamines, inflammation, immune substrate, and CMVD may improve risk stratification and lead to the development of targeted therapeutic strategies.

## 1. Introduction

Takotsubo syndrome (TTS) is a cardiomyopathy characterized by temporary left ventricular dysfunction that presents with kinetic abnormalities involving the apex (with the so-called “apical ballooning”, the most frequent form, found in 81.5% of the cases) and the mid-ventricular (~15%), basal (~2%) or focal segments (~1.5%), and sometimes the right ventricle [1,2]. Worldwide registries have estimated TTS to affect 1–3% of all patients and 5–6% of female patients with suspected ST-segment elevation myocardial infarction (STEMI) [3,4]. This incidence has been rising over the years (15–30 cases per 100,000 per year), although the true incidence is unknown as the condition is likely underdiagnosed [5].

The diagnosis of this condition is often complicated because the clinical presentation, electrocardiographic changes, and elevated indices of cardiac damage resemble those of acute myocardial infarction [1]. Only coronary angiography can rule out lesions affecting the epicardial coronary arteries, which could otherwise explain this acute phenomenon.

Usually, but not always, a physical, emotional, or combined trigger precedes the acute event; sometimes, the trigger can also be a neurological disorder (e.g., subarachnoid hemorrhage, stroke/TIA, or syncope) or pheochromocytoma. Patients who develop TTS following a neurological event present with a more nuanced clinical picture and no chest pain [6,7], whereas if the trigger is represented by emotional stress, chest pain and palpitations are more frequent [8]. The electrocardiogram typically shows repolarization abnormalities, including ST-segment elevation, T-wave inversion or depression, and QT interval prolongation; however, in rare cases, these changes are absent. A moderate increase in markers of cardiac damage (such as troponin and creatine kinase) frequently occurs, and a significant increase in cerebral natriuretic peptide is common [1].

The aim of this review is to provide an updated overview of the pathophysiological mechanisms underlying Takotsubo cardiomyopathy, with particular focus on the inflammatory and immune pathways and the major associated comorbidities. In addition, we summarize the main diagnostic approaches for detecting coronary microvascular dysfunction, aiming to improve the identification of higher-risk phenotypes. A better understanding of these underlying mechanisms may ultimately facilitate the development of more targeted therapeutic strategies.

## 2. Pathophysiology

The pathophysiological mechanisms underlying TTS are only partially understood; however, it is well established that sympathetic stimulation is central to the pathogenesis of the syndrome. In fact, the patterns of myocardial dysfunction typical of TTS can be replicated by intravenous administration of catecholamines and beta-agonists [9], and elevated concentrations of catecholamines can be found in the coronary sinus in patients with TTS [10].

Sympathetic function abnormalities affecting the myocardium may persist for months after recovery of left ventricular systolic function [11]. These abnormalities appear to induce interstitial mononuclear inflammation and occasionally the development of contraction band necrosis, a distinctive histological finding of the myocardium characterized by hypercontraction of sarcomeres, the presence of transverse eosinophilic bands, and a mononuclear interstitial infiltrate. This result appears dissimilar from the polymorphonuclear-mediated inflammatory damage seen in infarction [12], and coincides with the damage observed in clinical conditions such as pheochromocytoma [1].

### 2.1. Myocardial Stunning

Myocardial dysfunction in TTS is usually defined as myocardial stunning because it resembles typical post-ischemic stunning. Myocardial function normalizes rapidly after single ischemic episodes lasting less than 2 min; as the duration or severity of ischemia increases, there is a delay in the recovery of function despite the restoration of blood flow. Prolonged subtotal ischemia, as occurs in short-term hibernation, causes stunned myocardium despite restoration of perfusion, which may take a week to resolve if necrosis does not occur. Areas of stunned myocardium may coexist with irreversibly injured myocardium and contribute to the time-dependent functional improvements that follow myocardial infarction. Even though necrosis and pathological evidence of infarction are absent in the stunned myocardium, studies have shown that focal myocyte apoptosis and a concomitant increase in myocardial cytolysis indices are also part of the stunning mechanism [13].

Ischemic stunning generates damage through the release of free radicals (superoxide anion, hydrogen peroxide, and hydroxyl radical) and reduces calcium sensitivity of myofilaments, as demonstrated in experimental models in vitro and in vivo [13,14].

Temporary myocardial dysfunction in TTS is usually described as myocardial stunning because, similar to post-ischemic stunning, myocardial function remains depressed while resting myocardial perfusion is apparently normal. However, the molecular mechanisms underlying TTS differ significantly.

### 2.2. Etiological Hypotheses and Preclinical Evidence

The pathophysiology of this puzzling disease remains elusive, and the mechanism linking increased catecholaminergic stimulation to myocardial stunning is highly debated. In the early stages, it was thought to be caused by epicardial coronary spasm, a hypothesis that has been verified in isolated cases [15,16] but often rejected by invasive diagnostics [12]. The hypothesis of microvascular spasm was then advanced because abnormal coronary flow was found in patients with TTS (increased thrombolysis in myocardial infarction [TIMI] frame count) [17]; moreover, reduced coronary flow reserve and cardiac metaiodobenzylguanidine [MIBG] uptake defects on myocardial scintigraphy [18] suggest the presence of a sympathetic-mediated microcirculatory dysfunction. In fact, myocardial SPECT discloses a severe reduction in MIBG uptake, simulating denervation, in the normally perfused but dysfunctional segments during the acute phase of TTS, in overlap with the defects of myocardial glucose metabolism shown by PET [19,20]. Catecholamine damage is also thought to directly cause myocyte damage because elevated catecholamine levels reduce cardiomyocyte viability due to the accumulation of cyclic AMP-related calcium and oxygen-free radicals [14].

Whether coronary microcirculatory dysfunction plays a causative role or represents a secondary phenomenon triggered by myocardial inflammation and edema remains to be established [21]. An attempt to answer this question was made in a continuously monitored rat preclinical model in which no detectable perfusion defects preceded isoproterenol-induced apical ballooning, indicating that CMD is most likely a consequence rather than a cause of TTS [22]. Moreover, given that syndromes with symptoms of stable angina, non-obstructive coronary arteries, and invasive or non-invasive signs of microvascular dysfunction are classified as ischemic with non-obstructive coronary artery (INOCA), if coronary microvascular disease was a primary cause of TTS, patients with INOCA should be the most affected by TTS; however, this is not the case [23]. Furthermore, as explained below, most studies reporting on coronary microvascular dysfunction (CMVD) in patients with TTS during the index presentation reveal normalization of microvascular function during follow-up, parallel with normalization of ventricular wall motion abnormalities [24,25,26], corroborating the hypothesis that CMVD is a secondary phenomenon.

However, a significantly decreased endothelial function in a cohort of patients with previous TTS episodes was demonstrated through a flow-mediated vasodilatation test, the established and most reproducible method to evaluate it [27,28], providing the basis to attribute a causative role to CMVD. In the same cohort, no significant differences in arterial stiffness and intima–media thickness were present [27], suggesting a functional etiology of the syndrome.

#### 2.2.1. Inflammation

Inflammation likely plays a key role in the pathogenesis of this syndrome. Sampling and measurement of inflammatory biomarkers in the coronary sinus and aorta of patients with TTS compared to healthy subjects revealed increased markers of extracellular matrix remodeling and fibrosis; in the acute phase, interleukin (IL)-1 receptor antagonist and soluble T-cell immunoglobulin mucin domain-3 (sTIM-3) were more concentrated in the coronary sinus than in the aorta; growth differentiation factor-15 (GDF-15), osteoprotegerin (OPG), and von Willebrand factor (vWF) were more concentrated in both sites; and matrix metalloproteinase-9 levels were increased three months after the acute phase compared to healthy subjects [29].

Rats in which TTS-like cardiomyopathy was pharmacologically induced developed a characteristic inflammatory pattern. Early neutrophil infiltration was followed by clusters of myocardial macrophages. The proportions of M1 (proinflammatory tissue-destructive macrophages expressing iNOS and MHC class II) and M2 (CD163-positive anti-inflammatory, tissue-reparative/profibrotic macrophages) cells were examined. The number of M1 cells increased over time, peaking on day 4, whereas the number of M2 cells peaked on day 1 due to macrophage activation and decreased on day 7. There was a significant correlation between the percentage of M2 macrophages and the ejection fraction, suggesting that a preponderance of M2 improves recovery of cardiac function [30]. In rat models, global macrophage depletion (via clodronate liposome administration) or blockade of macrophage infiltration (via a CCR2 antagonist or in CCR2-KO mice) induced recovery after isoproterenol-induced TTS-like cardiac disfunction [31].

#### 2.2.2. Catecholamines and Calcium

The importance of catecholamines in the induction of TTS has been robustly demonstrated in vivo in preclinical rodent models. β_1_-adrenergic receptor (β_1_AR) signals through the canonical stimulatory G-protein (G_s_) pathway, whereas β_2_AR signals via G_s_ or G_i_ (inhibitory G-protein); adrenaline excess limits the inotropic and toxic effects of G_s_ by a shift in coupling to G_i_ in a process known as *stimulus trafficking*, which is enhanced by cyclic adenosine monophosphate-dependent phosphorylation of the β_2_AR [32]. An in vivo rat model is described in which intravenous epinephrine high-dose bolus produces apical depression with basal hypercontractility, and the effect is prevented via G_i_ inactivation by pertussis toxin pretreatment; in vitro isolated apical cardiomyocytes show greater number and functional response of β_2_AR compared to basal, and epinephrine induces direct cardiomyocyte depression in a β_2_AR-G_i_-dependent manner; this cardio-depressive effect at the apex is explained by the apical–basal gradient in β_2_Ars, and the G_i_ signaling (involving p38 MAPK and PI3K/Akt anti-apoptotic pathways) may have evolved as a protective mechanism to limit catecholamine-induced toxicity [32]. Sympathetic innervation is higher in the basal than in the apical myocardium, suggesting that the apical myocardium may be more sensitive to high blood levels of catecholamines released by the adrenal glands (mainly epinephrine) [33]. In healthy rodents the infusion of isoproterenol causes myocardial fibrosis, preferably in the apical segments [32,34].

Electrocardiographic changes in the acute phase of TTS are frequent; however, they often overlap with the myocardial infarction ECG criteria. T-wave inversion in 3–4 consecutive precordial leads is the most observed trait in the acute phase and may find an explanation in sympathetic hyperstimulation as well [35]. A direct effect on the myocardial conduction system by the sympathetic nervous system was suggested by studies on cats: it was found that stimulation of the right stellate ganglion increased T-wave amplitude, whereas the left stellate ganglion produced deeply inverted T-waves [36]; a greater effect on T-wave duration with left stellate ganglion stimulation was also described [37].

Several alterations of calcium metabolism have been reported in heart biopsies from patients with TTS within the acute phase; the SERCA2a (sarcoplasmic reticulum Ca-adenosine-triphosphatase 2a) gene expression is down-regulated while that of sarcolipin is up-regulated, and phospholamban is dephosphorylated, leading to reduced reticulum calcium reuptake, which contributes to both systolic and diastolic dysfunction [38,39]. Therefore, hemodynamic interactions should also be considered. It is hypothesized that epinephrine and norepinephrine-mediated vasoconstriction, increased reactive species generation, impaired mitochondrial function, lipid droplet accumulation, and higher NO levels produced by intense β_2_AR-G_i_ activation causes peroxynitrite generation leading to contractile dysfunction and inflammation; then, the interaction between the left ventricle and vascular afterload and inducible left ventricular outflow tract obstruction influences intracavity pressure gradients and generates myocardial dysfunction [40].

Catecholamines and endothelin exert their vasoconstrictor effects primarily in the coronary microcirculation, where α_1_ adrenergic receptors [41] and endothelin type A receptors are highly expressed, again suggesting the key role of microcirculatory dysfunction in TTS. Patients in the acute phase have been shown to present low levels of microRNA 125a-5p and high plasma levels of endothelin-1, its target, consistent with the etiological hypothesis of microvascular spasm [42]. Endothelin, moreover, represents one of the most powerful vasoconstrictors (100 times more powerful than noradrenaline) by accelerating the influx of calcium through voltage-gated calcium channels [43].

It is postulated that cases of isolated catecholaminergic stunning generally result in complete recovery with minimal residual inflammation or clinical complications. In contrast, the cases with combined catecholaminergic and ischemic stunning have greater inflammation and myocardial injury, and after the acute phase they are more likely to develop long-term complications due to persistent inflammation [39]. 

**Figure 1:**
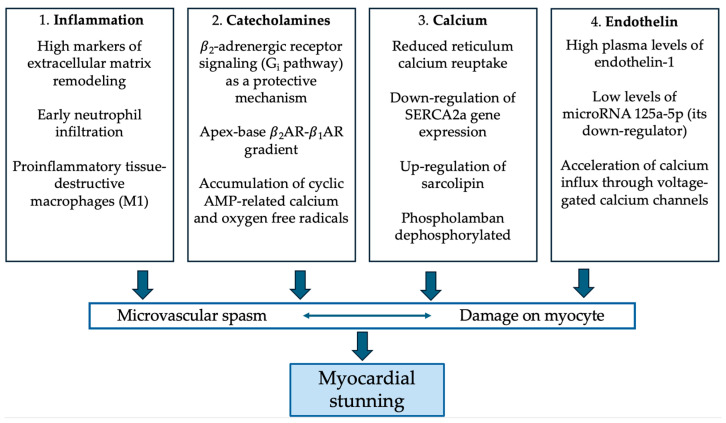
Molecular mechanisms contributing to the pathophysiology of TTS.

Figure 1 provides a summary of the main molecular mechanisms that lead to TTS.

## 3. Associated Predisposing Conditions, Risk Factors, and Predictors of Mortality

The predominance of postmenopausal women suggests that hormones may impact TTS. Women older than 55 years have an almost five-fold-higher risk of developing TTS compared to those younger than 55 [44]. Even if the exact link between estrogen levels and TTS has not been completely demonstrated, in women with subarachnoid hemorrhage, low levels of estradiol are associated with an increased risk of left ventricular wall motion abnormalities [45]. Furthermore, in ovariectomized rats subjected to a stress with contractile abnormalities, estrogen supplementation can attenuate the phenomenon [46].

Comorbidities associated with the development of TTS include hyperlipidemia, smoking, alcohol abuse, anxiety, and stress. In addition, it was found that hospitalization for TTS peaked in the summer months in contrast with AMI, which sees its peak during winter [44].

### 3.1. Genetic Factors

Although five cases of familial TTS have been reported [1], TTS does not appear to have a Mendelian inheritance pattern. More likely, a polymorphism in adrenergic genes interacts with the environment to increase susceptibility. Specific variants of adrenergic receptor genes have been associated with cardiac dysfunction in patients with subarachnoid hemorrhage [47] and pheochromocytoma [48], which can trigger TTS.

### 3.2. Psychiatric and Neurological Comorbidities

It has been verified that there is a higher prevalence of psychiatric and neurologic disorders in patients with TTS: 27% have an acute, former, or chronic history of neurologic disorders and 42% have psychiatric diagnosis, with half suffering from depression [2]. Moreover, anxiety and depression appear more common in TTS than in patients with STEMI or in healthy controls [49]. Notoriously, depressed patients have exaggerated norepinephrine response to emotional stress [50] and similarly, patients with diagnosed anxiety have a decreased catecholamine reuptake due to impairment of norepinephrine reuptake transporters [51]; this is also why selective norepinephrine reuptake inhibitors facilitate myocardial stunning by increasing local levels of catecholamines [52].

As previously mentioned, TTS frequently occurs after neurological disorders, especially stroke, subarachnoid hemorrhage, and seizures. Contraction band necrosis is a histopathological finding in autopsied patients who died unexpectedly of epilepsy [53], as well as after fatal TTS [54]. In particular, structural differences between TTS and healthy controls have been highlighted in the limbic network comprising the insula, amygdala, cingulate cortex, and hippocampus, all of which are areas strongly involved in emotional regulation [55]. Higher baseline amygdalar activity, assessed through 18F-FDG-PET/CT brain imaging, has been identified in a cohort of patients who subsequently developed TTS in comparison to matched controls and after adjusting for TTS risk factors; this heightened neurobiological activity exists even years before the onset of TTS and the highest levels correlate with an earlier onset [56]. This neural activity may represent a therapeutic target for reducing stress-induced diseases, including TTS.

### 3.3. Emotional and Physical Stressors

A distinctive feature of TTS is its association with a preceding stressful event. The typical patient is a postmenopausal woman who has experienced severe and unexpected emotional stress in the previous 1–5 days. While initially most reported triggers were emotional trauma, now we know that physical triggers are more common [2]. Male patients are more often affected by physically stressful events, while in women an emotional trigger can be more frequently observed [2].

A variety of physical stressors has been reported, including acute critical illness, respiratory failure, central nervous system disorders and iatrogenic factors such as surgery, exercise tests, or dobutamine stress echocardiography [57], and the presence of a physical stressor has also been associated with worse outcomes [58].

Diabetes mellitus is present in 10% to 25% of patients who experience TTS and is associated with increased mortality; it is thought that its impact on neuroautonomic nerve remodeling and up-regulation of vasoactive neuropeptides like NPY enhances susceptibility to stress cardiomyopathy [59]. Asthma exacerbation represents a possible trigger, mainly after medical interventions (short acting β_2_ adrenergic receptor agonist, epinephrine, and intubation) [60]. Malignancy also represents a frequent comorbidity [56,57].

Almost one third of TTS occurs without any evident trigger [2,57] and could be explained by considering the influence of psychosocial factors and pre-existing mental health comorbidities at the time of the event [5].

### 3.4. Autoimmune Disorders

Numerous case reports have demonstrated an association between acute TTS and chronic autoimmune diseases. A systematic scoping review of these cases showed an average age of 60 years and a strong female predominance with a female-to-male ratio of approximately 5.3:1. Among the autoimmune diseases of patients with TTS, myasthenia gravis was the most common, followed by systemic lupus erythematosus, multiple sclerosis, Guillain–Barre syndrome, Grave’s disease, and rheumatic arthritis; autoimmune thyroiditis, autoimmune polyglandular syndrome type II, vasculitis, and inflammatory bowel diseases were also fairly common [61].

Central immune tolerance is mediated by the thymus, where thymocytes mature into functional T cells. Thymocytes and early T cells that are self-reactive to cardiac antigens, such as myosin and troponin-I, generally undergo negative selection and apoptosis before entering the circulation. Nevertheless, a small percentage of autoreactive T cells can escape surveillance, resulting in circulating autoreactive T cells that possess the potential to react with exposed myocyte molecules under pathological conditions [62,63,64]. This mechanism has been recognized as a potential culprit of post-AMI or post-pericardiotomy pericarditis (Dressler syndrome). However, it is not yet known whether the same mechanism could partly explain the pathogenesis of TTS.

The dearth of reports demonstrating a link between autoimmune diseases and TTS is surprising, given that both conditions have a predilection for middle-aged women. A reasonable explanation is that patients with rheumatic conditions who develop TTS are already on various immunomodulating or immunosuppressive therapies that prevent a sufficient pro-inflammatory response to catecholamine peaks, potentially protecting them from the development of TTS [62]. Several studies have demonstrated that immune activation is triggered by acute myocardial infarction [65,66], and others have proposed the employment of known immunomodulators to reduce it (see, for example, the RITA-MI trial [67], a safety trial for the use of rituximab in STEMI). To date, no similar studies have been conducted on TTS. It must also be considered that TTS, myocardial infarction, and heart failure patients already receive similar pharmacotherapy, including anti-platelet therapy, β-blockers, statins, or ACE-inhibitors, that also affect the immune response, leaving room to speculate that some of their beneficial effects may act through immunomodulation [63].

Although suggestive, clear evidence establishing an association between autoimmune diseases and TTS remains lacking. Future studies should assess whether an autoimmune phenotype can predispose patients to the development of TTS, with a focus on autoimmune conditions that could contribute to the pathophysiology of TTS, such as inflammation and vasoconstriction. Recently, functional autoantibodies targeting angiotensin-II type 1 receptor (AT1R-AAs) and endothelin-1 type A receptor (ETAR-AAs) have been shown to play a relevant role in the context of STEMI [68]. AT1R and ETAR are G-protein-coupled receptors expressed on the surface of a variety of cells (immune cells, vascular smooth cells, endothelial cells, and fibroblasts), and their activation leads to inflammation, vasoconstriction, and fibrosis [69]. Given their presence in the general population and biological plausibility, future studies should investigate the relationship between these autoantibodies and microvascular disease in the context of TTS.

### 3.5. Predictors of Prognosis

The predictors of TTS prognosis remain largely unclear. The largest study that compares a cohort of STEMI patients to a similar TTS group evidences that TTS patients suffer from higher long-term mortality (24.7% versus 15.1% over one year) and, while mortality related to cardiovascular diseases (comprising myocardial infarction, heart failure, arrhythmia, sudden cardiac death, cerebrovascular disease, pulmonary embolism, or other vascular diseases) is similar, the difference is mainly explained by non-cardiovascular mortality, emphasizing the significance of comorbidities in TTS patients [70]. In a previous study, patients developing TTS in hospital after admission for other diseases consistently face higher mortality compared with patients admitted because of TTS [71]. The main independent predictors of mortality obtained through multivariable regression analysis were male sex, elevated Killip class on admission, and diabetes mellitus [70].

## 4. Diagnostic Methods to Assess Microvascular Damage

Currently, numerous methods are used to mark microvascular damage and the resulting increase in coronary microcirculatory resistance.

### 4.1. Non-Invasive Methods

Transthoracic echocardiography with pulsed Doppler allows the analysis of the coronary flow velocity ratio (CFVR) sampled in the anterior descending artery, which serves as a reference for the entire microcirculation. The ratio between the peak diastolic velocity of coronary blood flow at rest and during maximal hyperemia, obtained using adenosine or dipyridamole, correlates with microvascular disease. A CFVR value < 2–2.5 suggests microvascular dysfunction [72].

Positron emission tomography (PET) enables the quantification of myocardial blood flow at rest and during pharmacological stress (adenosine); the myocardial perfusion reserve (MPR, threshold value < 2) and the myocardial blood flow (MBF) can be evaluated [73].

Magnetic resonance imaging (MRI) using perfusion imaging allows the assessment of myocardial perfusion reserve (MPR), which acts as a surrogate for coronary flow reserve. Specifically, perfusion is proportional to the intensity of the T1 signal, which is determined by the diffusion of gadolinium from the microcirculation into the interstitial space [74]. T1 and T2 relaxation time and extracellular volume (ECV) are evaluated to characterize TTS in comparison to other conditions with similar clinical presentation [75]. Furthermore, CMR-feature tracking is used to determine circumferential strain (CS), radial strain (RS), and longitudinal strain (LS) to distinguish regional kinetic alterations typical of TTS with apical ballooning from those of acute myocardial infarction [76].

### 4.2. Invasive Methods

Microvascular function can be derived from coronary angiography. This method is based on the sequential selective coronary injection of 3 mL cold saline boluses, generally in the left anterior descending artery, under both resting conditions and after steady hyperemia induction. Using a pressure wire and a thermistor guide wire (PressureWire^®^, Abbott Laboratories, Abbot Park, IL, USA) at least 70 mm deep in the artery, it is possible to record the thermodilution curve at rest and in hyperemic conditions. The mean transit time at rest and during hyperemia is estimated, and surrogate indices of coronary flow and microvascular resistance can be derived. The most important are:

- Coronary flow reserve (CFR), derived from the ratio between the mean transit time at rest (Tmn_base_) and mean transit time in hyperemic conditions (Tmn_hyp_). It is pathological when ≤2;
- Index of microvascular resistance (IMR), derived from the product of distal coronary pressure in hyperemia (Pd_hyp_) and Tmn_hyp_, and considered pathological when >25;
- Resistive reserve ratio (RRR), calculated as the ratio between the basal resistance index (BRI = Pd_base_ × Tmn_base_) and IMR, and considered pathological when ≤2 [77].

The adoption of this method in clinical practice is limited globally because of its cost and the need for a dedicated pressure wire and steady-state hyperemia. To overcome these limitations, a wire-free method was developed to obtain similar results. Angiography-derived IMR is based on the application of computational flow dynamics to three-dimensional quantitative coronary angiography (QCA) and has been validated against invasive IMR. The main validated formula is the following:

$$NH-IMRangio=Pa\;\times \;cQFR\;\times \;\frac{Nframes}{fps}$$

where *Pa* is the mean aortic pressure, *cQFR* is the contrast quantitative flow-ratio (a computation derived from QCA analysis), *Nframes* is the number of frames for contrast dye to travel from the proximal reference to the distal one, and *fps* is the acquisition rate [78]. The shared cut-off to identify MVD is ≥25 in the setting of NSTEMI [79,80]. This formula has also been applied in TTS patients, even if in these patients the pathogenesis is more functional (endothelial dysfunction associated to the catecholaminergic cascade) rather than structural.

**Figure 2:**
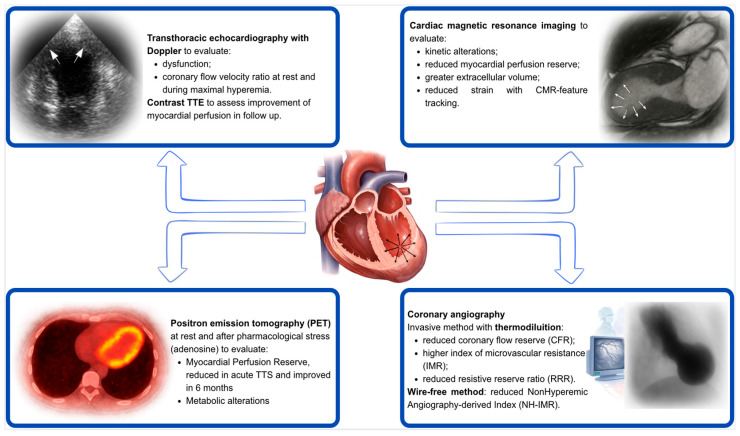
Main diagnostic tools to assess microvascular dysfunction.

Figure 2 summarizes the main diagnostic methods used to detect MMVD.

### 4.3. Current Evidence of Microvascular Damage in Takotsubo Syndrome

Coronary flow reserve (CFR), measured through color Doppler ultrasound of the anterior descending artery, is reduced in patients with acute TTS and manifests an early recovery parallel to functional recovery in regional wall motion, which remains substantially stable in the following six months [81]. In the acute phase, coronary flow velocity (CFV) during hyperemia correlates significantly with the wall motion score, that stands for systolic function, and with the indexed end-systolic volume, but not with E/Ea (the ratio between early diastolic transmitral velocity and average of septal and lateral mitral annulus early diastolic tissue velocity) as a parameter of diastolic function [25].

Contrast-enhanced echocardiography during adenosine infusion also reveals a marked improvement in myocardial perfusion and function in patients with TTS at baseline and after one month compared with a group of STEMI patients with a similar clinical presentation [25], proving a reversible microvascular vasoconstriction mechanism as a pathological mechanism underlying TTS, regardless of the trigger.

Cardiac magnetic resonance imaging has become a preferred non-invasive imaging modality for the diagnosis, prognosis, and follow-up of TTS [82]. Parametric tissue mapping revealed prolonged T1 and T2 relaxation times at 1.5T and 3T, respectively, with a higher extracellular volume fraction compared to healthy subjects and subjects with myocarditis, greater left ventricular end-diastolic and end-systolic volumes, and reduced global longitudinal strain [75]. CMR-feature tracking applied to TTS form with apical ballooning showed significantly reduced values compared to non-STEMI acute myocardial infarction patients and normal subjects; a global longitudinal strain ≤ 14.75 was also identified as a potential risk stratification parameter for long-term mortality. No significant difference in GLS values was detected between TTS and STEMI patients [76]. It was also proposed that, after TTS, patients who develop significant ECG pathological changes (e.g., ST-segment elevation followed by T-wave inversion and prolonged QT) and left ventricular disfunction suffer from transient myocardial edema, assessed by CMR, that could explain these changes. There is a significant linear correlation between the apicobasal ratio of T2-weighted signal intensity for myocardial edema and the amplitude of negative T-waves; repolarization changes appear unrelated to either late gadolinium enhancement or quantitative cine parameters [83].

Fluorodeoxyglucose PET rarely demonstrates perfusion defects in the acute phase of TTS, a pattern resembling myocardial stunning. Metabolism is frequently altered disproportionately to alterations in myocardial perfusion. It is hypothesized that in the acute phase of apical ballooning, a metabolic switch occurs from the use of fatty acid-free acids to anaerobic glycolysis; as previously mentioned, high levels of adrenaline trigger a switch in ventricular cardiomyocyte trafficking signals from stimulatory G_s_ proteins to inhibitory G_i_ proteins, resulting in a metabolic switch that has a protective purpose that this method is able to display [84]. In addition to demonstrating global microvascular dysfunction, PET is also useful in follow-up: myocardial blood flow (MBF) at rest and under stress and myocardial flow reserve are reduced during the acute event and improve at the six-month follow-up in all cardiac segments, as well as in patients with marked apical ballooning. However, abnormalities persist in the distal segments and at the apex, even in patients with full recovery of left ventricular function [85].

The non-hyperemic angiography-derived index of microcirculatory resistance (NH-IMRangio), as explained above, is a validated method for assessing coronary microvascular resistance. A positive index has proven to be highly correlated with TTS, particularly in the anterior descending artery distribution, and it is associated with diastolic dysfunction and BNP levels (but not troponin) [78,86].

Invasive microvascular dysfunction indices were used to highlight microcirculatory involvement in a cohort of patients with TTS in the acute phase compared with a similar cohort of patients with INOCA. The incidence of CMVD was higher in patients with TTS than in the INOCA cohort (78% versus 44%), with significantly higher mean IMR values and significantly lower CFR and RRR; the typical form with apical involvement showed greater microcirculatory involvement than the mid-ventricular form, regardless of the characteristics of coronary atherosclerosis assessed by IVUS [77]. Moreover, it has also been demonstrated that microvascular dysfunction can lead to impaired myocardial perfusion and, in more severe forms, impaired epicardial flow (slow coronary flow [SCF], defined as angiographically non-obstructive coronary arteries with TIMI-2 flow). Patients with coronary slow flow at admission have significantly higher occurrence of mortality and major cardiac events at long-term follow-up [87]. A case report shows that high IMR values of the acute phase normalize in the follow-up as left ventricular function recovers [24].

Table 1 summarizes the main studies addressing the investigation of microvascular dysfunction in TTS.

## 5. Therapeutic Options

Guidelines regarding TTS management are lacking because no prospective randomized trials have been performed in this specific patient population, resulting in therapeutic strategies based mainly on clinical experience and expert consensus. Despite the abundance of evidence supporting the role of microcirculatory disease in TTS, target therapy still remains elusive. The major objective of in-hospital treatment is to provide supportive care to accelerate recovery and minimize the worst complications.

Mild cases require no treatment or a short course of pharmacological therapy; in severe cases with acute heart failure and cardiogenic shock, early mechanical support should be considered as a bridge-to-recovery. Pharmacologic circulatory support such as adrenaline, noradrenaline, dobutamine, milrinone, and isoproterenol should be avoided [1], while levosimendan has recently shown to be safe and useful [88]. In the presence of left ventricular outflow tract obstruction, intravenous fluid and β_1_-selective beta-blockers should be considered, while diuretics and nitroglycerin should be avoided [1]. Conversely, if LF outflow tract obstruction is excluded, carvedilol should be prefered [39]. Angiotensin-converting enxyme inhibitors and beta-blockers can be used in the acute phase and then weaned once LV function has recovered, but could both be considered in recurrence prevention [1].

## 6. Future Perspectives

Although great progress has been made in understanding this multifaced condition, the literature still lacks data to interpret the wide variety of disease presentations and the diversity in both short- and long-term prognosis. Although current evidence indicates that microvascular damage is mostly acute-phase-related and apparently resolves with restitutio ad integrum, the underlying disease substrate that could clarify the most severe presentations, cases with residual long-term dysfunction and recurrent forms has yet to be demonstrated.

It is plausible that the answers to these questions lie in the coronary microcirculation, which we understand through indirect methods, but is still poorly understood in terms of its substance and structure. Moreover, considering the pathophysiology of TTS, with the predominant role of inflammation and vasoconstriction, it is conceivable that a specific autoimmune profile could also contribute to the development and prognosis of the syndrome.

Further studies to clarify these aspects would allow us to prevent the syndrome, treat it more effectively, and avoid the worst clinical outcomes.

## 7. Conclusions

Takotsubo syndrome is a complex clinical condition characterized by a close association between heart function and neurohormonal changes. The recognition of this acute heart failure syndrome has improved over the years with the awareness that it is not always a benign condition because of short- and long-term morbidity and mortality. Many aspects of TTS remain unclear, and the field of microvascular function is of particular interest in furthering our understanding of the syndrome and improving the quality of life and outcomes for affected patients.

## Figures and Tables

**Table 1:** Current diagnostic evidence of microvascular damage.

Diagnostic Tool	Evaluated Parameter	Studies
Transthoracic echocardiography	Coronary flow reserve through color Doppler ultrasound	Kume et al. 2005 [72] Rigo et al. (2010) [81]
	Contrast-enhanced echocardiography under stress	Galiuto et al. (2010) [26]
Cardiac magnetic resonance	Parametric tissue mapping	Humayra et al. (2024) [75] Ojha et al. (2020) [82]
	CMR-feature tracking	Stiermaier et al. (2018) [76]
	Apicobasal ratio of T2-weighted signal	Perazzolo Marra et al. (2013) [83]
Fluorodeoxyglucose PET	Perfusion abnormality	Yoshida et al. (2007) [54]
	Metabolic alterations	Testa et al. (2014) [20] Nayar et al. (2022) [84]
	Myocardial blood flow (MBF) at rest and under stress	Kadoya et al. (2025) [85]
Coronary angiography	Coronary flow reserve (CFR) Index of microcirculatory resistance (IMR)	Ekenbäck et al. (2023) [77] Warisawa et al. (2016) [24]
	Coronary slow flow	Montone et al. (2020) [87]
	Non-hyperemic angiography-derived index of microcirculatory resistance (NH-IMRangio)	Castaldi et al. (2023) [78] Scarsini et al. (2021) [80] Sans-Roselló et al. (2021) [86]

## Data Availability

No new data were created or analyzed in this study. Data sharing is not applicable to this article.

## Abbreviations

The following abbreviations are used in this manuscript:

ACE	Angiotensin-converting enzyme
AMI	Acute myocardial infarction
CMVD	Coronary microvascular dysfunction
CT	Computed tomography
FDG	Fluorodeoxyglucose
INOCA	Ischemia with non-obstructive coronary artery
iNOS	Inducible nitric oxide synthase
IVUS	Intravascular ultrasound
MAPK	Mitogen-activated protein kinase
MHC	Major histocompatibility complex
PET	Photon emission tomography
PI3K	Phosphoinositide 3-kinase
SERCA	Sarco/endoplasmic reticulum calcium ATPase
SPECT	Single-photon emission computed tomography
TTS	Takotsubo syndrome
